# Age at spinal cord injury determines muscle strength

**DOI:** 10.3389/fnint.2014.00002

**Published:** 2014-01-23

**Authors:** Christine K. Thomas, Robert M. Grumbles

**Affiliations:** ^1^The Miami Project to Cure Paralysis, University of Miami Miller School of MedicineMiami, FL, USA; ^2^Department of Neurological Surgery, University of Miami Miller School of MedicineMiami, FL, USA; ^3^Department of Physiology and Biophysics, University of Miami Miller School of MedicineMiami, FL, USA

**Keywords:** motoneuron death, muscle reinnervation, motor axon sprouting, muscle use, muscle fatigue, muscle strength

## Abstract

As individuals with spinal cord injury (SCI) age they report noticeable deficits in muscle strength, endurance and functional capacity when performing everyday tasks. These changes begin at ~45 years. Here we present a cross-sectional analysis of paralyzed thenar muscle and motor unit contractile properties in two datasets obtained from different subjects who sustained a cervical SCI at different ages (≤46 years) in relation to data from uninjured age-matched individuals. First, completely paralyzed thenar muscles were weaker when C6 SCI occurred at an older age. Muscles were also significantly weaker if the injury was closer to the thenar motor pools (C6 vs. C4). More muscles were strong (>50% uninjured) in those injured at a younger (≤25 years) vs. young age (>25 years), irrespective of SCI level. There was a reduction in motor unit numbers in all muscles tested. In each C6 SCI, only ~30 units survived vs. 144 units in uninjured subjects. Since intact axons only sprout 4–6 fold, the limits for muscle reinnervation have largely been met in these young individuals. Thus, any further reduction in motor unit numbers with time after these injuries will likely result in chronic denervation, and may explain the late-onset muscle weakness routinely described by people with SCI. In a second dataset, paralyzed thenar motor units were more fatigable than uninjured units. This gap widened with age and will reduce functional reserve. Force declines were not due to electromyographic decrements in either group so the site of failure was beyond excitation of the muscle membrane. Together, these results suggest that age at SCI is an important determinant of long-term muscle strength, and fatigability, both of which influence functional capacity.

## Introduction

In the United States, most traumatic spinal cord injuries occur in individuals who are 16–30 years old. However, more falls in older individuals have resulted in the average age at injury rising from 29 to 40 years over the last few decades (Jackson et al., 2004; NSCISC, 2013). Ostensibly, there are no differences in the numbers of intact axons or demyelination when traumatic human spinal cord injury (SCI) occurs at a young vs. old age (≥65 years, Furlan et al., 2010). Gains in sensory and motor scores were also independent of age during the first year after human SCI, the time when most recovery occurs (Ditunno et al., 1992; Waters et al., 1993; Kirshblum et al., 2004; Fawcett et al., 2007; Furlan and Fehlings, 2009; Pouw et al., 2011). Yet functional recovery was poorer in older individuals (≥65 years; Furlan and Fehlings, 2009).

Few animal studies have explored age-related differences after SCI. Like human studies, comparisons have largely been made between the young and the elderly. The results vary. Fiber tracts showed similar damage in young (2–3 months) and old rats (22–28 months) after partial dorsal hemisection (Jaerve et al., 2011). In contrast, pathology and demyelination were greater after spinal contusion, remyelination was reduced, and recovery of locomotor function was delayed in middle-aged (12 months) and old rats (24 months) compared to young rats (3 months; Siegenthaler et al., 2008). After thoracic cord hemisection, recovery of locomotor function was faster in young (40 days) and adult rats (60 days). Paw withdrawal was more frequent and faster in young (40 days) compared to adult (60 days) or middle-aged rats (12 months; Gwak et al., 2004). These latter results highlight functional differences occur early after SCI at young ages (40 and 60 days), drawing attention to descriptions of other age-related dysfunction after human SCI.

As people with SCI age, they report new muscle weakness, fatigue, and pain at ~45 years. These declines in physical function significantly impact independence, and quality of life. More assistance is needed with daily tasks (Gerhart et al., 1993; Kemp and Thompson, 2002; McColl et al., 2002; Amsters et al., 2005; Krause and Coker, 2006; Charlifue et al., 2010). The biological mechanisms underlying this late-onset impairment are unknown. Thus, we have no rationale strategy to address these aged-related declines in muscle function after human SCI. Is this muscle dysfunction at ~45 years impacted by the age at which SCI occurs? Or do ongoing changes in the years after injury (SCI duration) also contribute to this new muscle dysfunction?

Contusive SCI in humans damages axons and myelin. Grey matter is also destroyed at the injury epicenter (Bunge et al., 1993; Hayes and Kakulas, 1997; Guest et al., 2005), resulting in partial or complete muscle denervation (Peckham et al., 1976b; Thomas, 1997; Thomas et al., 1997; Mulcahey et al., 1999; Bryden et al., 2004; Kern et al., 2005). Survival of both motoneurons and interneurons is important to retain muscle innervation and use, both of which influence muscle strength, fatigue, and ultimately function (Needham-Shropshire et al., 1997; Thomas and Zijdewind, 2006). In this study, our aim was to make a cross-sectional analysis of paralyzed thenar muscle and motor unit contractile properties in two datasets obtained from different subjects who sustained a cervical SCI at different ages. Examining the extent of muscle weakness when SCI occurs at a younger vs. young age provides some indication of the importance of motoneuron death on muscle force (Thomas and Zijdewind, 2006) since near elimination of activity reduces forces to 40–50% initial (Tower, 1937; Pierotti et al., 1991). We have also compared thenar strength and fatigue in relation to age at the single motor unit level. This approach shows how well intact motoneurons function independently of confounds that impact muscle strength (e.g., motoneuron death).

## Methods

The whole muscle and motor unit data presented here have been compiled from our published studies. Only a synopsis of the methods is given. All of the experiments were approved by the University of Miami Investigational Review Board. All subjects gave written informed consent before participation in the experiments.

### Setup and protocol for thenar muscle force and EMG measurements

All subjects sat in a chair or wheelchair with the test arm resting to the side and supported in a vacuum cast (Thomas, 1997). The hand rested in modeling clay, palm up, and was held in place by a metal plate and Velcro. A transducer was aligned with the thumb and used to measure abduction and flexion forces at right angles. Resultant force was calculated. EMG data were also recorded from the distal and proximal muscle surfaces using wire electrodes taped across the entire muscle (Westling et al., 1990). The median nerve was stimulated just proximal to the wrist using pulses of increasing intensity (1–10 mA steps) until maximal compound muscle action potentials (M-waves) were evoked (no further increase in EMG with an increase in stimulus intensity). The maximal force was then evoked by 1 s of stimulation at 50 Hz using a supramaximal stimulation intensity (20–50% greater than the level that produced a maximal M-wave). Force and EMG were sampled online at 400 Hz and 3200 Hz, respectively, using a SC/Zoom system (Umeå University, Sweden).

**Figure 1:**
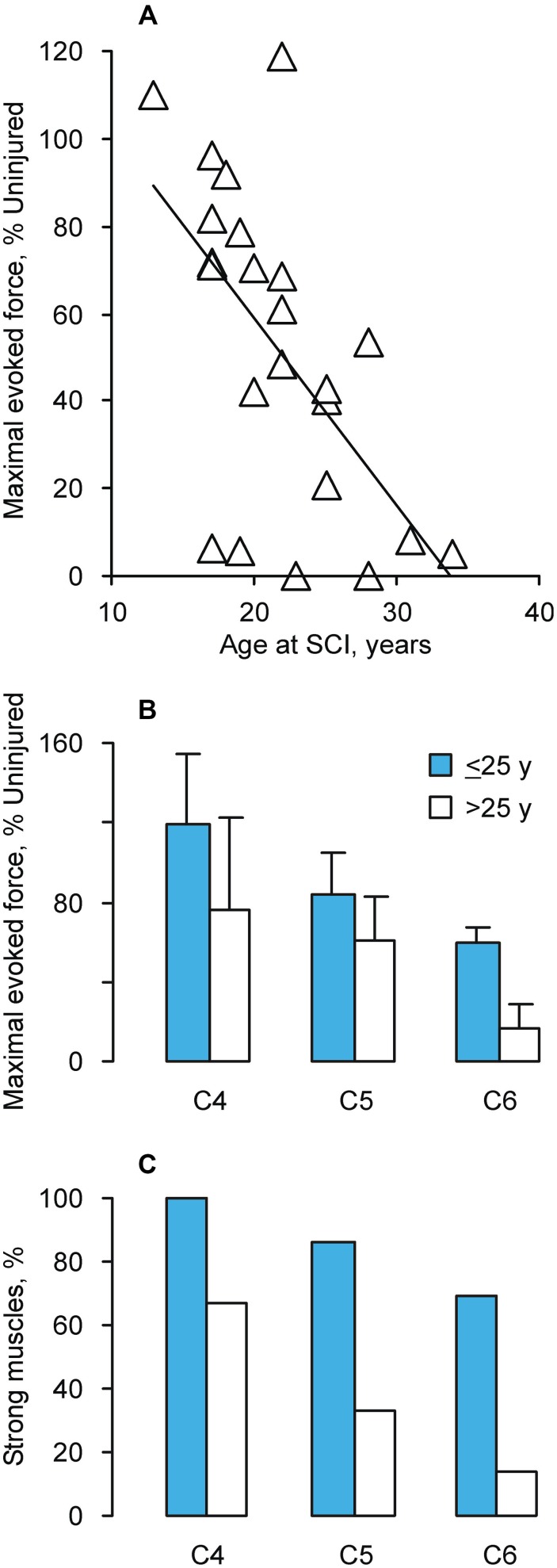
**(A)** Maximal evoked force vs. age at which C6 SCI occurred (*n* = 23 muscles; *y* = −4.28*x* + 145.07, *R*^2^ = 0.36). Mean (+SE) forces for uninjured 20 and 30 year old men were 26.7 ± 1.6 N and 22.9 ± 4.2 N, respectively. The corresponding data for women were 16.4 ± 1.7 N and 18,7 ± 1.8 N. **(B)** Mean (±SE) maximal evoked thenar force (*n* = 42 muscles) normalized to age and sex-matched uninjured data (*n* = 40 muscles). **(C)** Percentage of strong muscles (>50% uninjured force), separated by SCI level and age at SCI (Younger: ≤25 years; Young: >25 years; *n* = 42 muscles).

Maximal thenar muscle forces (baseline to peak) were measured using Zoom software. Data were obtained from 42 paralyzed muscles. These people had SCI at C4 (*n* = 6), C5 (*n* = 13) or C6 (*n* = 23) due to a diving accident (*n* = 18), a motor vehicle accident (*n* = 14), gunshot (*n* = 4), a sports accident (*n* = 5) or a fall (*n* = 1; Thomas, 1997; Zijdewind and Thomas, 2001; Griffin et al., 2002; Thomas et al., 2003; Butler et al., 2004). Results were separated into two groups according to whether the person had been ≤25 years (termed, Younger) or >25 years (termed, Young) at the time of SCI, respectively. This separation was used because maximal evoked thenar force was 38% uninjured at 25 years (Figure 1), close to the reduction expected for an inactive muscle (Tower, 1937; Pierotti et al., 1991). Table 1 lists the mean (±SE) age at SCI and SCI duration by injury level and age group. Maximal forces from paralyzed muscles were normalized to data obtained from 40 muscles of uninjured subjects matched by age and sex.

**Table 1:** **Mean (±SE) age by SCI level and group**.

Group	Younger			Young		
SCI level	C4	C5	C6	C4	C5	C6
Age at SCI, years	22 ± 2	21 ± 1	20 ± 1	39 ± 1	31 ± 2	30 ± 1
SCI duration, years	8 ± 2	9 ± 2	11 ± 2	6 ± 2	4 ± 2	6 ± 2

### Thenar motor unit counts

The experimental arrangement and data collection were the same as described for muscle force and EMG measurements but the protocol differed. Five pulses at 1 Hz were delivered to the median nerve at increasing intensities (0.1 mA steps) from subthreshold (no response) to supramaximal intensity. Responses with similar EMG and force were averaged. The averaged data was ranked from weak to strong force and overlayed. The number of force steps was used as an estimate of the motor unit count. Data were obtained from six men who sustained a SCI at C5 (*n* = 1), C6 (*n* = 3) or C7 (*n* = 2) from a diving accident (*n* = 3), a motor vehicle accident (*n* = 2) or a fall (*n* = 1). Age at SCI and SCI duration ranged from 21–46 years and 3–15 years, respectively (Thomas et al., 2002).

### Thenar motor unit forces

Unit forces were recorded in response to intraneural stimulation of thenar motor axons in the median nerve (Westling et al., 1990). Each person reclined on a bed with the test arm and hand resting to the side. Both the unitary EMG and force were recorded as described for whole muscle measurements. Maximal motor unit force was evoked by stimulation at 30, 40, or 50 Hz for 1 s or at 100 Hz for 0.5 s. Fatigue was induced by delivering 13 pulses at 40 Hz each second for 2 min. The force fatigue indices were calculated every 20 s as the ratio between the current force and the initial force. EMG area fatigue indices were calculated similarly using the first potential of the respective stimulus trains. Data were obtained from 12 individuals with SCI (Häger-Ross et al., 2006; Klein et al., 2006; Thomas et al., 2010) at C4 (*n* = 4), C5 (*n* = 3) or C6 (*n* = 6) from gunshot (*n* = 1), a diving accident (*n* = 3), a motor vehicle accident (*n* = 5), horseback riding (*n* = 2) or a fall (*n* = 1). Data were divided by age, as described for whole muscle forces. Age at SCI averaged (±SE) 19 ± 1 years for the younger group, and 34 ± 3 years for the young group, respectively. The corresponding values for SCI duration were 10 ± 2 years, and 9 ± 2 years, respectively. These data were compared to that obtained from 12 uninjured subjects (23 ± 1 years and 39 ± 7 years for the younger and young groups, respectively).

### Statistics

Mean (±SE) data are given. The relationships between maximal evoked force and age at C6 SCI, and between muscle force and motor unit counts were analyzed using least squares linear regression. The proportion of weak vs. strong muscles was compared using Chi square analysis. Two-way ANOVA was used to compared mean muscle force in relation to age at SCI and level of SCI, as well motor unit force in relation to group (SCI vs. Uninjured) and age. Motor unit force and EMG area fatigue indices were examined over time using two way repeated measures ANOVA.

## Results

### Paralyzed thenar muscles were stronger when younger individuals sustained high level spinal cord injury (SCI)

The maximal evoked thenar force declined when C6 SCI occurred in older individuals (*p* = 0.003, Figure 1A). The regression analysis showed that the expected force would be 59%, 38%, and 17% of uninjured values when SCI occurred at 20, 25, and 30 years of age, respectively. For comparison, the mean maximal thenar muscle force for 30 year old uninjured men (22.9 ± 4.2 N) was 85% of the mean measured for 20 year old men (26.7 ± 1.6 N). There was no significant relationship between evoked force and SCI duration (*p* = 0.42) or current biological age (*p* = 0.45).

The maximal evoked force from completely paralyzed thenar muscles was also significantly lower when the SCI was closer to the thenar motor pools (*p* = 0.035, C6 vs. C4) and almost significant when the person was younger when injured (≤25 years, *p* = 0.052, Figure 1B). To estimate the prevalence of muscle strength vs. weakness, these same data were separated by force. More muscles were strong (>50% uninjured force) if the person was younger when injured, irrespective of injury level (*p* < 0.001, Figure 1C).

### Motoneuron survival maintained muscle strength

**Figure 2:**
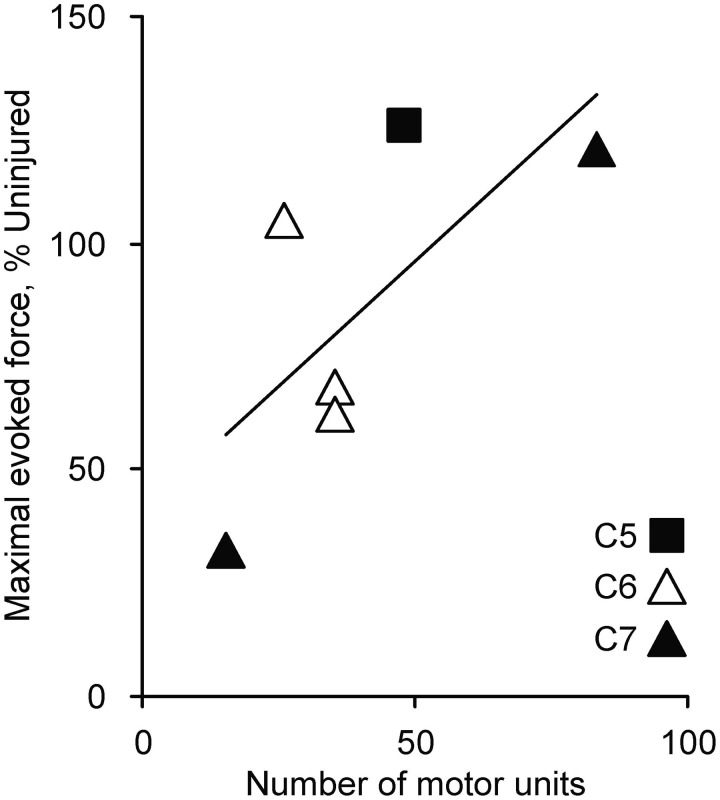
**Number of motor units in relation to maximal thenar muscle force evoked by supramaximal median nerve stimulation and expressed relative to uninjured means (*n* = 6 subjects)**.

The number of thenar motor units was correlated positively with maximal force (*p* = 0.118, *n* = 6 muscles, Figure 2). Although not significant, 70% of the force variability was accounted for by motor unit number. Motoneuron survival is therefore an important determinant of muscle force. Reductions in thenar motor unit numbers occurred in each SCI case (range: 15–83 units) since estimates for uninjured individuals averaged 144 ± 16 (Yang et al., 1990; Thomas et al., 2002). Only ~30 units survived each C6 SCI (~20% uninjured) with maximal muscle force ranging from 62% to 105% of uninjured values. Since intact axons only sprout 4–6 fold (Brown et al., 1981), the limits for muscle reinnervation have largely been met in these individuals who ranged in age from 25–53 years, when studied. Any further reduction in motor unit numbers with time after these injuries will likely result in chronic denervation, which is one factor that may explain the late-onset muscle weakness routinely described by people with SCI.

### Thenar motor units were less fatigable when spinal cord injury (SCI) was sustained at a younger age

**Figure 3:**
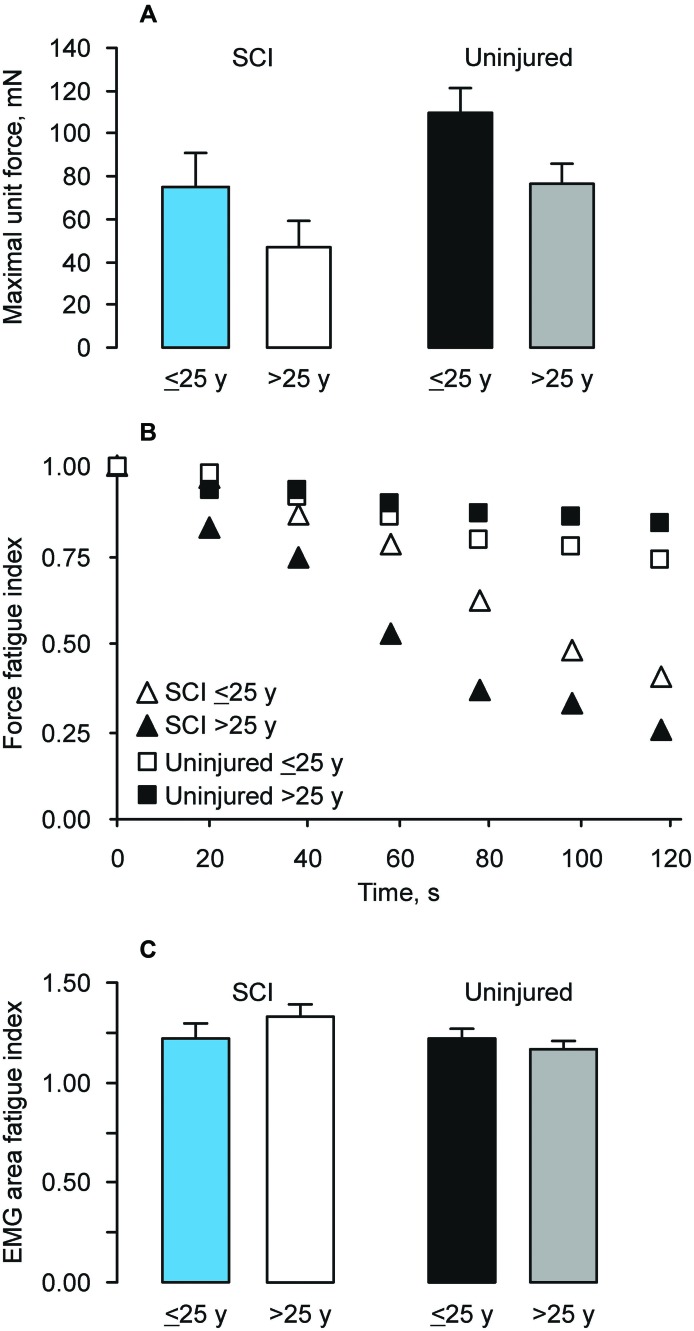
**(A)** Mean (+SE) maximal thenar motor unit force for subjects injured at a younger (≤25 years) and young (>25 years) age compared to uninjured subjects. **(B)** Relative decline in force (current value/initial value) in response to 13 pulses at 40 Hz every second for 2 min by group and age at SCI. **(C)** Relative changes in EMG area after 2 min of stimulation, as described in **B**, by group and age at SCI.

The initial mean maximal force of paralyzed thenar motor units was weaker than that for units of uninjured subjects (*p* = 0.019; Häger-Ross et al., 2006). Motor unit forces were also weaker with age (*p* = 0.023), but there was no interaction between group and age (Figure 3A). Paralyzed motor units from individuals injured at a younger age generated 68% of the force produced by units from age-matched uninjured people. The corresponding value for young SCI subjects was 62%.

In contrast, paralyzed motor units were more fatigable than uninjured units and the gap widened with age. Mean force declines (fatigue) differed by group (*p* < 0.001) and time (*p* < 0.001, Figure 3B). The time effects also differed among groups (*p* < 0.001). After 2 min of stimulation, paralyzed motor units from individuals injured at a younger age had fatigue indices that were 44% lower than units from age-matched uninjured people, whereas the fatigue indices were 70% lower for individuals injured when they were young. Force declines reached significance at 20 s for the young SCI group, at 40 s for the younger SCI group, at 60 s for the younger uninjured group, and at 80 s for the young uninjured group. Force declines were greater for the young SCI group compared to both uninjured groups (from 40–120 s) and the younger SCI group (from 60–120 s). The younger SCI group fatigued more than both uninjured groups from 80–120 s. The younger uninjured group fatigued more than the young uninjured group at 120 s. However, the EMG area increased significantly (*p* < 0.001) from 20–120 s, suggesting that the force declines were distal to excitation of the sarcolemma (Figure 3C; Klein et al., 2006). The mean EMG area fatigue indices recorded from the young SCI group were higher than those from both uninjured groups (*p* = 0.006) but there was no interaction between group and time, possibly because larger increases in both EMG potential duration and area occur in more fatigable units (Klein et al., 2006; Thomas et al., 2006).

## Discussion

Our data show that age at SCI impacts both muscle strength and motor unit fatigue, even in young adults. After SCI at C6, maximal thenar force was reduced to 59% of uninjured if the person was 20 years old. Force was 42% lower (17% uninjured) when the SCI occurred at 30 years of age. No relationship was evident between force and the duration of SCI. These data indicate that age at the time of SCI has a strong influence on recovery of muscle strength. At the single motor unit level, age weakened unit forces in both SCI and uninjured subjects. However, paralyzed units were more fatigable, especially in people injured at >25 years of age. Greater muscle fatigue may be shaped by time-dependent changes.

### Muscle strength depends on innervation

The compressive and/or contusive forces that commonly occur with human SCI destroy grey matter at the lesion epicenter, kill motoneurons, and denervate muscles (Bunge et al., 1993; Hayes and Kakulas, 1997; Thomas and Zijdewind, 2006). Compensatory mechanisms exist when the denervation is incomplete. Intact axons are challenged to sprout within muscle to innervate up to 4–6 times more muscle fibers (Brown et al., 1981; Yang et al., 1990; Thomas et al., 1997). Thus, if only a few motoneurons die, intramuscular sprouting and reinnervation will resolve any muscle strength deficits. However, force deficits do become evident when 70–80% of motoneurons die. The limits of axon sprouting are met, leaving some muscle fibers denervated chronically.

Our data show that most individuals who sustained SCI when they were older than 25 years had forces below 50% uninjured, particularly when the SCI was close to the test motor pools (Figure 1). Motoneuron survival was also an important determinant of muscle strength (Figure 2). These novel data raise many questions about the recovery of muscle function after SCI. Do more motoneurons die when SCI occurs in a person older than 25 years, inducing greater muscle weakness? Does age influence the robustness of intramuscular sprouting? Does the sprouting response in muscle differ if a young vs. older motoneuron has lost or retained most of its synaptic inputs after SCI? To our knowledge, none of these questions have been explored in relation to age at SCI. The more extensive paralysis that occurred in adults vs. children with poliomyelitis suggests an age-related susceptibility of motoneurons to death in this situation (Weinstein, 1957; Jubelt et al., 1980). Sprouting of central axons is compromised in old (22–28 months) vs. young rats (2–3 months) after partial dorsal hemisection (Jaerve et al., 2011). Recovery of muscle function is better after peripheral nerve section in children compared to young adults (Rosen and Lundborg, 2001; Chemnitz et al., 2013; Galanakos et al., 2013). More frequent and faster paw withdrawal after spinal hemisection in young (40 days) compared to adult (60 days) and middle-aged rats (12 months; Gwak et al., 2004) suggest that early responses to SCI change recovery of muscle function in younger animals. Clearly, new studies are needed to define the relevance of these results to recovery of muscle function when SCI occurs in different aged humans.

### Muscle strength and fatigue depend on use

Ongoing neural activity due to spasticity, continuous ongoing activity in the relaxed state, changes in muscle length, and loading may be sufficient to counter atrophy in some paralyzed muscles (e.g., Figure 1B, Younger C4 SCI; Thomas, 1997). However, reduced amounts of daily activity will contribute to muscle weakness. Our data show that motor units were weaker with paralysis and with age, but the force declines were independent of group (SCI vs. Uninjured; Figure 3A). Motor units in uninjured hand muscles are activated at low intensities each day (Kern et al., 2001; Thomas et al., 2005). Ongoing spontaneous motor unit activity is also common in paralyzed thenar muscles and unit firing rates are low (Stein et al., 1990; Zijdewind and Thomas, 2001, 2012). Thus, activity may be less important than spinal pathology in determining age-related differences in muscle strength after SCI. Strength declines from chronic use of baclofen (Thomas et al., 2010) are less likely to explain force differences because 58% and 54% of the units in the younger and young SCI datasets were influenced by this medication, respectively. Both SCI groups also included data from individuals with C4, C5 or C6 SCI, minimizing effects from injury level.

Fatigability of paralyzed units increased with age, whereas motor units were less fatigable in young uninjured adults (Figure 3B). The different force declines did not reflect failure of neuromuscular transmission because potentiation of EMG area with repeated stimulation was a consistent finding across groups irrespective of age (Figure 3C; Klein et al., 2006). With time, the proportion of fast type fibers increases in paralyzed muscles, as does their fatigability, largely due to reductions in muscle activity (Peckham et al., 1976a; Stein et al., 1992; Burnham et al., 1997; Shields and Dudley-Javoroski, 2006; Thomas and Zijdewind, 2006). If greater destruction of spinal circuitry occurs with SCI and age, it may be harder to activate affected muscles, increasing their fatigability. In healthy muscles, the least active motor units are the most fatigable ones (Hennig and Lømo, 1985; Thomas et al., 1991; Klein et al., 2010).

### Spinal cord injury (SCI) leaves many muscles close to the threshold for dysfunction

Another striking feature of our data is the wide variation in strength of completely paralyzed muscles (Figure 1). Each of these muscles would receive a manual muscle score of zero so clinical evaluations miss these age-related force differences completely. Knowledge of paralyzed muscle force generating capacity is important when patterned electrical stimulation is to be used to restore function to these muscles, an approach which people with SCI value (Brown-Triolo et al., 2002; Anderson, 2009), but this strategy would only be valuable for paralyzed muscles that can generate strong enough forces for useful function. In contrast to the data presented here, the reports of new muscle dysfunction at ~45 years probably arise from muscles that are weakened, but left under voluntary control by SCI. In muscles under voluntary control, manual muscle scores can range from 1 to 5 but any given manual muscle score is associated with a wide range of voluntary forces. In triceps brachii for example, the average force associated with a manual muscle score of 3, which is considered useful for function because the test joint can be moved through its full range of motion against gravity, ranged from 2–19% of the maximal voluntary force of uninjured subjects. Force ranged from 0–16% of maximal force for a score of 2 (full elbow extension without gravity), so for some muscles, shortening was felt and they generated EMG but this activity resulted in no measurable force (Needham-Shropshire et al., 1997). These data show that the voluntary forces measured in triceps brachii after SCI overlapped for different scores, making it important to question whether the low resolution of manual muscle scores (5 possible levels) has limited the ability to discern age-related differences in voluntary muscle strength. This is even more of an issue when published data are pooled across many muscles rather than presented for each muscle.

Our data show that SCI at a young age (≤40 years) leaves many muscles near the threshold for clinical symptoms (Figure 1; 10–20% uninjured force; McComas et al., 1993). Both the weakness and fatigability of paralyzed muscles will limit performance in applications involving patterned electrical stimulation because more muscle has to be used to generate a given force. Only small decrements in maximal force after injury or from fatigue would result in meaningful declines in muscle function. Thus, age at SCI is a risk factor that likely affects early onset (~45 years) muscle weakness and fatigue after SCI. But the underlying mechanisms are unknown. Obviously, trauma of the spinal cord will perturb multiple factors and pathways essential for neuron survival and plasticity of local spinal and neuromuscular circuits. Intrinsic neuron survival and axon growth are developmentally regulated, and generally become less robust in early adulthood. After injury, less axon regeneration occurs from mature neurons than young ones (Filbin, 2006). Thus, SCI at an older age may immediately result in less optimal neuroprotection and retention of spinal circuits.

It is also conceivable that slow changes over many years induce late-onset muscle dysfunction after SCI. This may involve alterations in the original pathology. For example, syrinx development can take several years (Brodbelt and Stoodley, 2003). After age 40, activity-based force declines average 1.5% per year in the uninjured (Clement, 1974; Frontera et al., 1991; Lauretani et al., 2003; Frederiksen et al., 2006) so weakness may reflect less muscle use. Alternatively, regressive changes in motor unit function may be attributable to chronic overuse of weak muscles that have been partially denervated by SCI. In poliomyelitis, residual motor units are used excessively (Borg et al., 1988), but as these individuals age motor unit numbers unexpectedly decrease at up to twice the rate that occurs in healthy subjects (McComas et al., 1997; Grimby et al., 1998; Gonzalez et al., 2010). Ongoing cycles of denervation and reinnervation occur in muscles of people with post polio syndrome (Hayward and Seaton, 1979; Borg et al., 1988; Grimby et al., 1998) so chronic intramuscular axon growth in over exerted motor units could also undermine motor unit integrity. Traumatic SCI involves additional central damage compared to that which occurs with poliomyelitis. Thus, greater central dysfunction and peripheral demands after SCI and age may elevate calcium levels in motoneurons to excess, resulting in mitochondrial dysfunction, damage and death (van den Bosch et al., 2006; Gleichmann and Mattson, 2011; Jacobs et al., 2013). Motoneuron survival with age may therefore rely on managing metabolic load in relation to the growth demands of reinnervated motor units. Consistent with this idea, partial muscle denervation in rats 3 weeks after botulinum toxin-induced muscle paralysis resulted in more motoneuron death than botulinum toxin or partial denervation alone (White et al., 2000). Following nerve crush, unexpected motoneuron death also occurred in mice overexpressing GAP43, which causes constant sprouting (Harding et al., 1999).

### The impact of age at spinal cord injury (SCI) on muscle function is understudied

We lack substantive evidence on how spinal pathology, muscle innervation (motor unit number, force), and muscle use change with SCI and age. Our data emphasizes that this information is needed throughout the lifespan, not just in the young and the elderly. All of these processes are important determinants of strength and how well a muscle can be used for daily tasks. These unresolved issues are key questions to answer for the aging SCI population because muscle contractions define functional independence and other body functions (e.g., blood glucose levels, bone strength; Vandervoort, 2002; Pedersen, 2009). This kind of information is needed to facilitate design of interventions to remedy age-related declines in muscle function after SCI. If muscle weakness is primarily determined by early motoneuron survival, sustained neuroprotection has to become a priority. If overuse of surviving motoneurons predisposes them to death, as suggested with post polio syndrome, exercise has to be tailored for long-term muscle health.

## Author contributions

Christine K. Thomas: Project conception and design, data acquisition, analysis, and summary, review of literature, manuscript writing. Robert M. Grumbles: Project conception and design, review of literature, manuscript writing.

## Conflict of interest statement

The authors declare that the research was conducted in the absence of any commercial or financial relationships that could be construed as a potential conflict of interest.
